# Retraction: Molecular structure and dimeric organization of the Notch extracellular domain as revealed by electron microscopy

**DOI:** 10.1371/journal.pone.0335095

**Published:** 2025-10-27

**Authors:** The PLOS One Editors

Following publication of this article [1], the corresponding author TW contacted Harvard Medical School regarding concerns about the results in this article. A review by Harvard Medical School concluded that the results in Figs S1 and 1D are unreliable and inconsistent with the underlying data. Specifically, the review found that:

- Whilst the Fig S1 legend states that the images represent averages of 6917 particle images classified into 20 classes, the underlying data for Fig S1 shows that the Fig S1 images were derived from three parent classification sets, one set containing 70 classes, one set containing 80 classes, and one unknown set of images.
- Some of the published Fig S1 images appear to be identical to images in the set of 70 classes and in the set of 80 classes.

Regarding the similarities between the underlying data and the Fig S1 images, first author DFK stated that the original dataset of over 6917 particle images was refined through selectively culling images with low signal to noise ratio, then regrouping the remaining images. This process was repeated and included the parent classification sets of 80 classes and 70 classes, until the final subset of 20 classes in Fig S1 was obtained. The review by Harvard Medical School found that this methodology does not account for the similarities observed between the Fig S1 images and the subsets of images containing 70 classes and 80 classes.

In addition to the concerns above, the *PLOS One* Editors noted similarities between the Fig S2B Drosophila data and the Fig S3B human data. Specifically, the Fig S2B top row column 3 panel appears similar to the Fig S3B top row column 6 panel with vertical flipping, and the Fig S2B bottom row column 9 panel appears similar to the Fig S3B bottom row column 3 panel with rotation.

First author DFK stated the underlying data for Fig S2B and Fig S3B are no longer available. They stated that the above panels in Fig S2B and Fig S3B are not identical and similarities in the white features of the panels are accounted for by the similar size between the Drosophila and human proteins. A member of the *PLOS One* Editorial Board reviewed these figures and the first author’s response, and stated that they remain concerned about similarities between these figures. PLOS does not consider these concerns to be resolved in the absence of the underlying data.

In light of the above concerns that question the reliability of the reported results, the *PLOS One* Editors retract this article.

RJL, TCM, HYF, and SAT agreed with the retraction. TW agreed with the retraction and apologizes for the issues with the published article. DFK did not agree with the retraction.
